# General hospital patients’ attitude towards systematic health risk behavior screening and intervention

**DOI:** 10.1186/s12889-024-20410-2

**Published:** 2024-10-18

**Authors:** Caroline Timm, Filipa Krolo-Wicovsky, Anika Tiede, Marie Spielmann, Beate Gaertner, Ulrich John, Jennis Freyer-Adam

**Affiliations:** 1 Institute for Medical Psychology, University Medicine Greifswald, Walther- Rathenau-Str. 48, D-17475 Greifswald, Germany; 2 German Centre for Cardiovascular Research e.V, Partner site Greifswald, Fleischmannstr. 42- 44, D-17475 Greifswald, Germany; 3 Department of Epidemiology and Health Monitoring, Robert Koch Institute Berlin, General- Pape-Str. 62-66, D-12101 Berlin, Germany; 4 Institute for Community Medicine, Department Prevention Research and Social Medicine, University Medicine Greifswald, Walther- Rathenau-Str. 48, D-17475 Greifswald, Germany

**Keywords:** Health behavior, Health risk factors, Behavior change counseling, Patient approval

## Abstract

**Background:**

Systematic counseling on behavioral health risk factors (HRFs) may be suitable to promote health among general hospital patients. This study aimed to investigate the openness of patients towards systematic screening and intervention of HRFs, its relation to actual participation in a multi-behavioral intervention, and whether socio-economic characteristics, HRFs and health indicators are related to approval.

**Methods:**

All 18- to 64-year-old patients hospitalized in five medical departments at the University Medicine Hospital Greifswald in Germany were asked between May and July 2022 to participate in a survey and in a subsequent pre-post intervention study. Among all eligible patients, 225 (78.9%) participated in the survey. Patients’ approval of systematic screening and intervention of HRFs was assessed using five statements with a total sum score of 0–20 (i.e., scores of 0–6, 7–13, 14–20 referring to low, medium and high approval). Associations with intervention participation, socio-economic, behavioral and health-related patient characteristics were analyzed using logistic and multivariable linear regression analyses.

**Results:**

The mean total approval of screening and intervention was 13.8 (SD = 4.8). Of the 125/ 73/ 16 patients with high/ medium/ low approval, 88.0%/ 78.1%/ 50.0% participated in the subsequent intervention, respectively. Approval was independent of socio-demographic and -economic characteristics and self-rated general health. Current tobacco smoking was the only HRF negatively (*p* = 0.02) and diabetes mellitus was the only disease positively (*p* = 0.01) associated with approval.

**Conclusion:**

High approval of HRF screening, which was rather independent of socio-economic characteristics and worse self-rated general health, speaks in favor of proactively approaching and motivating all general hospital patients to participate in health behavior change intervention. Tobacco smokers might need higher efforts to motivate participation than non-smokers.

**Trial registration:**

ClinicalTrials.gov Identifier NCT05365269 on May 9, 2022.

## Background

About 90% of the adults in western countries exhibit one or more behavioral health risk factors (HRFs), including tobacco smoking, at-risk alcohol use, overweight, insufficient vegetable and fruit intake and physical inactivity [1–3]. About half of the adult general population in Germany and other western countries report two or more co-occurring behavioral HRFs [3, 4], with even greater proportions (66%) among general hospital patients [5]. Behavioral HRFs have an impact on global morbidity and mortality [6, 7], and each additional HRF significantly increases the risk for mortality [6, 8]. To prevent and to support the treatment of non-communicable diseases effectively, behavior change counseling in health care is recommended [9, 10]. Systematic screening is a proactive measure to reach as many individuals of the target population with as little selection as possible. Reach is one of the crucial dimensions achieving high public health impact of behavioral interventions [11]. Annually about 16% of the German general adult population are admitted to a hospital [12]. Thus, general hospital patients might be a target group that is particularly appropriate for screening of behavioral HRFs and brief intervention. Hospitalization itself and the worry about the current condition might be a “window of opportunity” for behavioral HRF screening and intervention given easy access and the teachable moment [13, 14]. However, little is known about three issues that are relevant in this case: the overall approval rate among hospital patients, whether socio-economic characteristics affect the approval, and whether approval might be limited to those who are generally in poor health or affected by a non-communicable disease or by particularly high load of behavioral HRFs. In the UK, among general hospital patients who were questioned post-discharge, 80% agreed with screening and 87% rated the hospital an ideal setting to receive health education [15]. However, given the participation rate of 59%, these encouraging findings might be biased by having been derived from rather selective sample of previous patients with particularly high motivation to change behavioral HRFs. Nevertheless, research has shown that medical staff or intervention providers often underestimate patients’ motivation to change alcohol use or to get counseling [16]. In contrast, 66% of the German patients are open towards counseling as found for at-risk alcohol use [17], and 96% of primary care patients in the USA would appreciate advice when drinking is affecting their health [18]. People with characteristics indicating lower socio-economic position such as a low level of school education, are harder to reach for behavior change interventions than people with higher levels of school education [19]. It remains unclear whether this might be explained by lower approval of such measures in patients with a low level of school education or who are unemployed. By using a proactive recruitment approach in the hospital, this social inequity might be reduced at least. It might be plausible that poor general health and the occurrence of non-communicable disease might affect the approval of behavioral HRF screening and counseling in the way that those with worse health might be more open towards screening and counseling because they already feel threatened by the disease.

We intended to investigate general hospital patients’ approval of systematic behavioral HRF screening and intervention and its relation to actual participation in a multi-behavioral intervention. Furthermore, we intended to investigate whether patient characteristics such as socio-demographics and -economics as well as behavioral HRFs and current health status are associated with the approval of systematic screening and intervention.

## Methods

### Sampling frame and participants

Data from the pre-post-intervention study “Proactive automatized lifestyle intervention for cancer prevention: Pilot-test (PAL-Pilot)” were used [20]. The data protection officer and the ethics committee of the University Medicine Greifswald approved the study (BB 024/17; BB 024/17a).

**Fig. 1:**
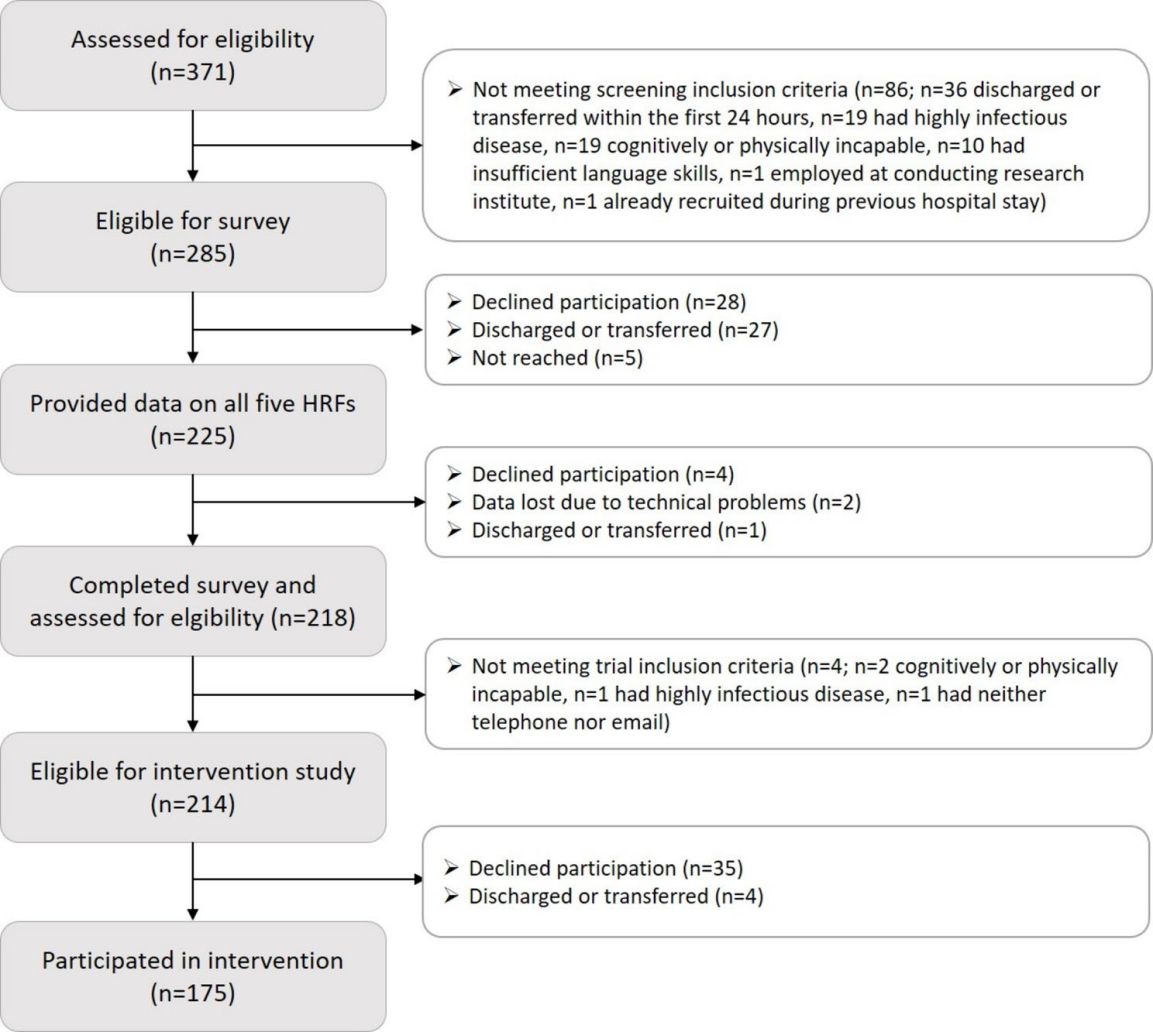
Patient flow for survey and intervention participation

Over six consecutive weeks between May and July 2022, participants were systematically recruited at the University Medicine Hospital Greifswald in Germany. The recruitment took place on 11 wards in five major medical departments (general surgery, trauma surgery, otorhinolaryngology, internal medicine, with the internal medicine departments including gastroenterology, endocrinology, nephrology, cardiology, angiology and pneumology). On Tuesdays through Fridays, all patients aged 18–64 years and submitted the days before were approached by a research assistant and asked to fill in a survey on health risk behaviors using tablet computers. As depicted in more detail in Fig. 1, of 371 admitted patients, 86 were excluded due to not meeting the screening inclusion criteria. Those 285 patients eligible for the survey were asked to provide informed oral and electronic consent for participation in the survey in a first step. Of these, 225 (78.9% of those eligible) participated and provided data on all behavioral HRFs for the current study.

In a second step, those 218 participants who finished the survey completely, were checked for eligibility to participate in the subsequent longitudinal pre-post intervention study. Four completers were not eligible for trial inclusion criteria. At this time, the patients were unaware whether any behavioral HRFs were present, and the study staff was unaware of patient survey responses. Of those 214 eligible patients, 175 participated in the pre-post study. The multi-behavioral intervention PAL-Pilot, as described in more detail elsewhere [20], involved highly individualized and computer-based feedback based on the preceding assessment data and on behavior change theory, delivered through three computer-generated feedback letters at three time points; i.e. directly after baseline, and one and three months later.

### Measurements

Data were derived from the baseline PAL-Pilot survey which is fully and in more detail described elsewhere [20].

Approval of systematic health risk behavior screening and intervention was assessed using five statements. Each of these is rated from 0 (strongly disagree) to 4 (strongly agree), similar to a previous study [15]. Approval of systematic screening of behavioral HRFs was assessed using four items, one each on tobacco smoking (“All patients should be asked about their tobacco smoking”), alcohol use (“All patients should be asked about their alcohol use”), eating behavior (“All patients should be asked about their vegetable and fruit intake”) and physical activity (“All patients should be asked about their physical activity”). Approval of behavior change intervention was assessed using the item “All patients should be informed if their behavior could contribute to the development of diseases or interfere with treatment”. All five items were summed up to a total approval score ranging from 0 to 20. Internal consistency was good with Cronbach’s alpha = 0.89. The total approval score was categorized respectively as low (0–6), medium (7–13), and high (14–20) total approval based on the same range. Participants were also asked to report their preferred way of delivery of individualized lifestyle feedback with three response categories, i.e. online/ email, by regular letter or undecided.

Participation in intervention was determined by participants’ entry into the intervention program targeting behavioral HRFs as part of the subsequent pre-post-intervention trial (yes/ no).

Socio-demographics included age in years, sex (men/ women) and partner status (yes/ no). First partner status was explored by asking “What is your current family status?” with five response options (single/ married/ married but separated/ divorced/ widowed). Non-married participants were further asked “Do you currently live in a relationship?” (yes/ no). Those married or living in a relationship were considered to be in a partnership.

Socio-economic characteristics involved the assessment of years of school education and employment status. School education was categorized into low (< 10 years), medium (10–11 years) and high level (> 11 years). Unemployment status (yes/ no) was assessed by asking “Are you currently employed?” with three response options (yes, full-time/ yes, part-time/ no). Participants responding “No” were asked which of eight response options applied (student, unemployed < 6 months, unemployed > 6 and < 2 years, unemployed > 2 years, housewife or househusband, military or voluntary service, maternity leave, retired). Those with any length of current unemployment were considered unemployed. All other responses were considered as not unemployed.

Five behavioral HRFs were assessed. Any current daily or occasional tobacco smoking was considered as HRF, which was estimated by asking “Do you currently smoke?” with four response options (current daily smoking/ occasional smoking/ former smoking/ never smoking). At-risk alcohol use was determined using the Alcohol Use Disorder Identification Test-Consumption [21], with cut-off values of ≥ 4 for women and ≥ 5 for men indicating at-risk alcohol use [22]. Overweight was determined using the body-mass-index measured by self-reported weight in kilograms and height in meters and the formula body weight (kg)/ height (m)^2^, with values of ≥ 25 indicating overweight [23]. Insufficient vegetable and fruit intake was determined using the question “How many servings of vegetable and fruit do you eat on average per day?” and twelve serving examples such as one medium-sized carrot or apple. Servings < 5 indicated insufficient vegetable and fruit intake [24]. Physical inactivity was assessed using an adapted version of the European Health Interview Survey-Physical Activity Questionnaire [25]. For walking, cycling as part of every-day life activities and sports, the number of minutes spend per typical week was determined. To determine whether the recommendations of the World Health Organization [26], i.e. at least 150 min of moderate (or 75 min of vigorous, or a respective combination of both) physical activity per week are met, three additional items, asking whether participants breathe or sweat harder or whether their heart beats faster, were used to discriminate between no, light, moderate and vigorous activity. To calculate minutes of moderate physical activity, the response categories were no/ yes, often/ yes, always for walking and cycling; and according minutes were multiplied by 0/ 0.5/ 1, respectively. For sports, response categories were a little stronger/ very much stronger/ differing from time to time; and reported minutes were multiplied by 1/ 2/ 1.5, respectively. Less than 150 min per week of moderate and/ or less than 75 min per week of vigorous physical activity was considered insufficient.

Health status was assessed by the presence of the four most common non-communicable disease groups (cancer, cardiovascular diseases, chronic respiratory diseases, diabetes mellitus type I or II or gestational diabetes) and self-rated general health. Each of the four disease groups was assessed by the question “Have you ever been diagnosed by a doctor with [cancer/ cardiovascular disease/ chronic respiratory disease/ diabetes mellitus]?”. Examples of diseases were provided for cardiovascular disease (hypertension, myocardial infarction, coronary heart disease, stenocardia, myocardial insufficiency, heart failure) and chronic respiratory disease (chronic bronchitis, chronic obstructive pulmonary disease, pulmonary emphysema). All responses indicating a diagnosis during the current hospital stay, within the past 12 months, or more than 12 months before the current hospital stay were considered as the respective disease being present. General health was measured using the question “How would you rate your own health in general?” with responses on a linear scale from 1 (poor) to 5 (excellent). This item is a reliable and independent predictor of mortality [27].

### Statistical analysis

Descriptive information is given for sample description. To determine the approval of systematic screening and behavior change intervention, the number of cases (N) and proportions (%) among eligible patients were evaluated per item, and the mean total approval score was determined (M, SD). To investigate screening and intervention approval as a predictor of actual participation in a subsequent behavior change intervention, logistic regression analysis was used and odds ratios (OR) and 95% confidence intervals (CI) were calculated. To identify patient characteristics as predictors of patients’ approval, a multivariable linear regression with all available and potentially relevant socio-demographic and -economic (i.e. age, sex, partner status, level of school education, unemployment status), behavioral HRFs (i.e. tobacco smoking, at-risk alcohol use, overweight, insufficient vegetable and fruit intake, physical inactivity) and health-related predictors (i.e. non-communicable diseases, general health) was used and ß-coefficients were calculated. Cases with missing values were excluded listwise. Statistical significance was tested with *p* < 0.05. Power calculation revealed that the sample size of *n* = 214 was sufficient to identify medium sized effects of d = 0.5 (80% power, α = 0.05, two-tailed) between two independent groups of different sizes (e.g. n_1_ = 175, n_2_ = 39). Stata version 17.0 was used for all analyses [28].

## Results

### Sample characteristics

The mean age of all 225 health survey participants was 49.8 years (SD = 12.7), the age range was 18 to 64 years. A total of 126 (56.0%) participants were men, 143 (63.6%) had a medium level of school education, 170 (75.6%) were living in a partnership and 14 (6.2%) were unemployed, 117 (53.9%) reported at least one non-communicable disease (Table 1). The mean general health score was 2.7 (SD = 0.9). Almost all participants (99.6%) reported at least one of the five behavioral HRFs. The mean number of behavioral HRFs was 2.8 (SD = 1.0).

**Table 1:** Sample characteristics

Variable	*N*	%
**Socio-demographics and –economics**	225	100.0
Sex		
Men	126	56.0
Women	99	44.0
Partner status		
In partnership	170	75.6
Not in partnership	55	24.4
Level of school education		
Low	36	16.0
Medium	143	63.6
High	46	20.4
Unemployment status		
Unemployed	14	6.2
Not unemployed	211	93.8
**Present behavioral health risk factors (multiple responses)**	225	100.0
Tobacco smoking (yes)	79	35.4
At-risk alcohol use (yes)	66	29.3
Overweight (yes)	148	65.8
Insufficient vegetable and fruit intake (yes)	212	94.2
Physical inactivity (yes)	127	56.4
**Present non-communicable disease (multiple responses)**	217	100.0
Cancer (yes)	28	12.9
Cardiovascular disease (yes)	79	36.4
Chronic respiratory disease (yes)	30	13.8
Diabetes mellitus (yes)	32	14.8

### Approval of systematic screening and behavior change intervention

The total score of patients’ approval of systematic screening and intervention for HRF ranged from 0 to 20 as theoretically possible; and the mean was 13.8 (Table 2). Of all health survey participants, 58.4% (125) reported high, 34.1% (73) medium, and 7.4% (16) low approval. Concerning systematic screening for each single HRF, approval mean scores ranged between 2.6 for physical activity and 2.8 for tobacco smoking. The mean approval of systematic behavioral intervention for HRF was 2.9. Ninety participants (40.2%) reported to prefer lifestyle feedback by regular letter, 85 (37.9%) by email or online, and 49 (21.9%) were undecided.

**Table 2:** Patients’ approval rated between 0 (“strongly disagree”) to 4 (“strongly agree”)

Variables	*N*	M	SD	Observed range
Approval of systematic screening for tobacco smoking	224	2.84	1.16	0–4
Approval of systematic screening for alcohol use	224	2.74	1.17	0–4
Approval of systematic screening for vegetable and fruit intake	224	2.70	1.10	0–4
Approval of systematic screening for physical activity	224	2.63	1.06	0–4
Approval of behavioral intervention	224	2.90	1.18	0–4
Total score of approval of systematic screening and behavior intervention	224	13.81	4.76	0–20

### Approval and subsequent participation in behavior change intervention

**Fig. 2:**
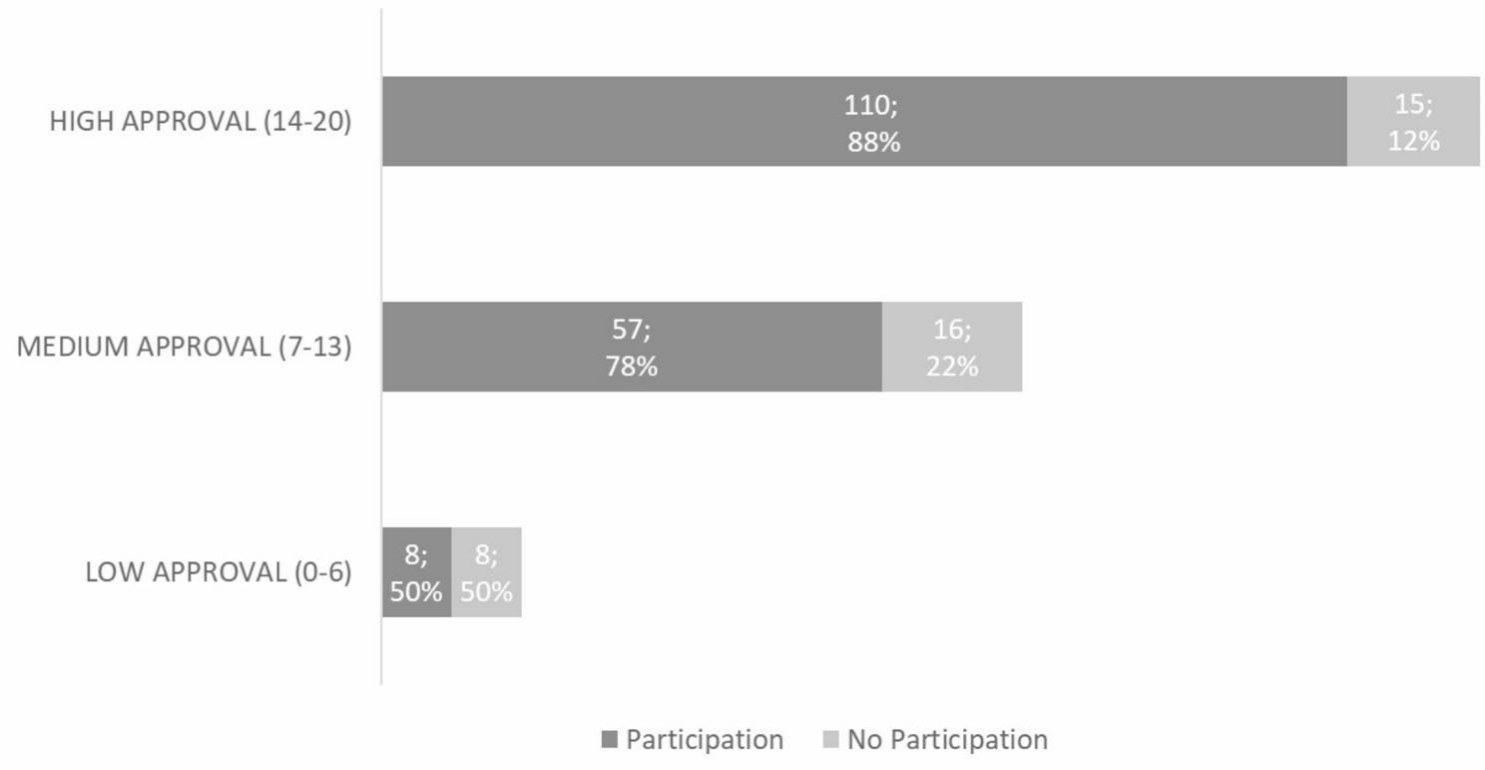
Patient participation in brief intervention stratified by total approval score (n;%)

Among the 214 patients eligible for the intervention trial, the total score of approval of systematic screening and intervention for HRF was positively associated with actual participation in the subsequent intervention. The likelihood of participating in the intervention increased by 11% for each of the 20 ranks of the total acceptance scale (odds ratio = 1.11; 95% confidence interval = 1.04–1.20; *p* = 0.021). As depicted in Fig. 2 and 88.0% (110/125) of the patients with high approval, 78.1% (57/73) of the patients with medium approval, and 50.0% (8/16) of the patients with low approval participated in the subsequent intervention.

### Associations with total approval score

Socio-demographic and -economic variables, most behavioral HRFs, most diseases and general health were not significantly related to the total approval score of behavioral HRF screening and intervention in a multivariable linear regression analysis (Table 3). Tobacco smoking was negatively related to the total approval score (β=-1.7; *p* = 0.02). Diabetes mellitus was positively related to total approval (β = 2.6; *p* = 0.01).

**Table 3:** Predictors of total approval score of systematic screening and intervention for health risk factors (multivariable linear regression, *N* = 214)

	β	*p*
**Socio-demographics and -economics**		
Age in years	-0.05	0.099
Women versus men	-0.82	0.209
Not in partnership versus in partnership	-0.46	0.539
Medium versus low level of school education	1.00	0.291
High versus low level of school education	1.34	0.248
Unemployed versus not unemployed	-2.53	0.067
**Behavioral health risk factors**		
Tobacco smoking (yes versus no)	-1.70	0.019
At-risk alcohol use (yes versus no)	-0.62	0.394
Overweight (yes versus no)	0.03	0.964
Insufficient vegetable and fruit intake (yes versus no)	0.15	0.911
Physical inactivity (yes versus no)	0.04	0.950
**Health status**		
Cancer (yes versus no)	1.13	0.268
Cardiovascular disease (yes versus no)	-1.34	0.061
Respiratory disease (yes versus no)	1.71	0.071
Diabetes mellitus (yes versus no)	2.57	0.007
General self-rated health	0.21	0.590

## Discussion

This study has three key findings. First, 58% of the general hospital patients reported high approval of systematic behavioral HRF screening and intervention. Second, approval turned out to be independent of socio-economic patient characteristics. Third, approval was independent of health in general and non-communicable diseases, such as cancer or cardiovascular disease.

The satisfying proportion of patients who approved behavioral HRF screening and intervention confirms that general hospital patients are open towards health behavior change intervention, as reported in previous studies [15, 17]. Furthermore, our findings suggest that screenings provided proactively, with personal contact with each patient, can successfully reach patients, not only those with high approval of screening and intervention. Even among those with low approval, 50% participated in the subsequent intervention. In terms of public health impact of interventions [11], systematic approaches may reach large parts of the target groups, including significant proportions of those patients with initially low or medium approval. Using new information technologies like computer-based interventions may support the dimension of reach as they may be distributed to a greater number of patients at lower costs [29], and may help tackle implementation barriers such as high workload and limited time [16]. Furthermore, the approval turned out to be strongly associated with participating in the intervention. Each of the 20 ranks of the total approval scale was associated with an 11% higher likelihood of participating in the intervention. Our results suggest that proactive recruitment and high levels of approval are a promising combination to get many patients into intervention. The approval of systematic screening and brief intervention turned out to be independent of socio-economic status and further sociodemographic characteristics. This is important as interventions show considerable selection bias in self-selected samples. That is, often those with low socio-economic status and who have been shown to be particularly affected by behavioral HRFs [5] are less well reached by behavioral interventions than those with high socio-economic status [19]. Our findings suggest that systematic screening and intervention in general is suited to decrease such social inequality in terms of approval. However, small effects regarding unemployment may not have been detected due to insufficient samples size.

Our findings suggest that behavioral HRF screening and intervention is approved both by those who suffer from poor health or from disease and by those who do not. This finding stands in favor of screening and brief intervention being suited for the entire range of people according to their health. The findings concerning the lower approval of patients who smoke tobacco is in line with previously reported findings on feeling uncomfortable or judged when reporting on their smoking habits [30]. Greater social pressure and stigmatization of tobacco smoking, particularly in health care settings, may be experienced. Interestingly, at-risk alcohol use, another often stigmatized behavioral HRF [31], was not related to lower approval in our study. We suspect that in contrast to tobacco smoking, patients are not as much aware of their level of alcohol use being considered a health-risk. The finding that patients with diabetes disclosed greater approval than patients without diabetes might be due to their high engagement and compliance to disease management-programs [32, 33] and them already being used to regular screening and behaviour counselling [34, 35]. Altogether, the findings indicate that while the mere presence of behavioural HRFs is not or negatively related to approval, the presence of diseases as diabetes and also found by trend for chronic respiratory disease, is positively related to approval.

### Limitations and strengths

A few limitations of this study should be noted when interpreting the findings. First, as the study was based on self-report, responses may be biased due to the tendency to provide socially desirable answers [36]. Self-report could lead to over- or underestimation of HRFs, as has been found for under-reporting of overweight, for example [37]. However, self-report is the foundation of behavior change interventions and interventionists can only work with what the individuals are willing to disclose. The selected self-report measures have good predictive validity of clinically relevant outcomes and whenever possible, valid assessment instruments like the Alcohol Use Disorders Identification Test-Consumption [21] were used. Second, given that 22% of the eligible patients did not participate in the survey, their approval or disapproval is unknown. Among those who explicitly declined to participate in the survey, we did not assess the reasons for decline, whether it may have been e.g. data safety concerns or disapproval of health surveys. However, given the proactive approach of the study, one crucial strength of this study was that the information was obtained for a high proportion of patients (79%) with lower selectivity of the sample in comparison to previous studies [15]. Third, our sample is restricted to hospitalized patients. Nevertheless, similar results of patients’ approval of systematic health risk behavior screening and intervention might be expected in primary medical care.

## Conclusion

The data of this study suggest that approval of systematic health risk behavior screening and intervention is high among general hospital patients, that it is independent of socio-economic status characteristics and independent of whether the patient is in particularly poor health or not. The findings speak in favor of systematic health risk behavior screening among general hospital patients as a first step in motivating patients to change health risk behavior.

## Data Availability

The dataset analyzed during the current study is not publicly available due to the German data protection law but are available from the principle investigator of the study, Prof. Dr. Jennis Freyer-Adam on reasonable request that complies with the study purpose and the participants’ informed consent.

## Abbreviations

β: Beta coefficient
HRFs: Health risk factors
M: Mean
n: Number of cases
PAL-Pilot: Proactive automatized lifestyle intervention for cancer prevention: Pilot-test
p: P-value
SD: Standard deviation
